# Obesity in adolescents with chronic fatigue syndrome: an observational study

**DOI:** 10.1136/archdischild-2016-311293

**Published:** 2016-09-21

**Authors:** T Norris, K Hawton, J Hamilton-Shield, E Crawley

**Affiliations:** 1 School of Social & Community Medicine, University of Bristol, Bristol, UK; 2 NIHR Bristol Biomedical Research Unit in Nutrition and University of Bristol, Bristol, UK; 3 Centre for Child and Adolescent Health, School of Social and Community Medicine, University of Bristol, Bristol, UK

**Keywords:** ALSPAC, Obesity, CFS/ME, Observational

## Abstract

**Objective:**

Identify the prevalence of obesity in patients with chronic fatigue syndrome (CFS) compared with healthy adolescents, and those identified with CFS in a population cohort.

**Design:**

Cross-sectional analysis of multiple imputed data.

**Setting:**

Data from UK paediatric CFS/myalgic encephalomyelitis (CFS/ME) services compared with data collected at two time points in the Avon Longitudinal Study of Parents and Children (ALSPAC).

**Patients:**

1685 adolescents who attended a CFS/ME specialist service between 2004 and 2014 and 13 978 adolescents aged approximately 13 years and 16 years participating in the ALSPAC study.

**Main outcome measures:**

Body mass index (BMI) (kg/m^2^), sex-specific and age-specific BMI Z-scores (relative to the International Obesity Task Force cut-offs) and prevalence of obesity (%).

**Results:**

Adolescents who had attended specialist CFS/ME services had a higher prevalence of obesity (age 13 years: 9.28%; age 16 years: 16.43%) compared with both adolescents classified as CFS/ME in ALSPAC (age 13 years: 3.72%; age 16 years: 5.46%) and those non-CFS in ALSPAC (age 13 years: 4.18%; age 16 years: 4.46%). The increased odds of obesity in those who attended specialist services (relative to non-CFS in ALSPAC) was apparent at both 13 years (OR: 2.31 (1.54 to 3.48)) and 16 years, with a greater likelihood observed at 16 years (OR: 4.07 (2.04 to 8.11)).

**Conclusions:**

We observed an increased prevalence of obesity in adolescents who were affected severely enough to be referred to a specialist CFS/ME service. Further longitudinal research is required in order to identify the temporal relationship between the two conditions.

**What is already known?**

- Chronic fatigue syndrome/myalgic encephalomyelitis (CFS/ME) and obesity are important long term health problems. However, little is known about the relationship between CFS/ME and obesity in adolescents.
- Physical activity and sedentary behaviour may be important mechanisms by which CFS/ME and obesity are associated however, research to date has provided conflicting results.

**What this study adds?**

- At 13 years, adolescents who had received a diagnosis of chronic fatigue syndrome/myalgic encephalomyelitis (CFS/ME) who were attending specialist CFS/ME services were more than two times more likely to be obese than adolescents in the general population.
- At 16 years, they were more than 4 times more likely to be obese compared to those in the general population.

## Introduction

Little research has been conducted investigating the relationship between chronic fatigue syndrome/myalgic encephalomyelitis (CFS/ME) and obesity, especially in adolescents. The few cross-sectional studies which have been conducted have provided conflicting findings,^1–4^ while a large longitudinal study found no association between obesity at 10 years and self-reported CFS diagnosis by the age of 30 years.^5^

Obesity is an important comorbidity as it contributes an important risk for the development of a range of metabolic disorders (eg, type II diabetes, dyslipidaemia, hypertension and coronary heart disease).^6–9^

Adolescents with CFS/ME may have an increased susceptibility for the development of obesity, via decreased levels of physical activity and/or increased time spent doing sedentary activities^3^ ^10^ and it is plausible that greater risk will be observed in those with more debilitating CFS/ME. As obesity has been observed to track from youth into adulthood,^11^ ^12^ maintaining the associated increased risk for metabolic sequelae across the life course, it is important to identify whether a diagnosis of CFS/ME in adolescence is associated with the prevalence of obesity, so appropriate interventions can be implemented.

The objective of this study was to obtain prevalence estimates for obesity at two time points during adolescence (ages 13 years and 16 years), in three groups of adolescents representing a continuum of CFS/ME severity (healthy population, CFS/ME based on responses to questionnaires and clinically diagnosed CFS/ME).

## Methods

### Sample

Participants used in this study came from two sources. First, we use data collected from all National Health Service (NHS) paediatric specialist services that participated in the CFS/ME National Outcomes Database (NOD) between August 2004 and October 2014. During this period, clinical assessment data and patient-reported measures were collected for approximately 1700 children and young people.

Second, to represent the epidemiological arm of the study, we use data from adolescents participating in the Avon Longitudinal Study of Parents and Children (ALSPAC) cohort. ALSPAC is a population-based longitudinal birth cohort of children who had an expected date of delivery between April 1991 and December 1992 and whose mothers were resident in the Avon region of South-West England at the time of recruitment.^13^ From 14 541 pregnancies included, 13 978 children were alive at 12 months of age (excluding triplets and quads) (Please note that the study website contains details of all the data, ie, available through a fully searchable data dictionary: http://www.bris.ac.uk/alspac/researchers/data-access/data-dictionary/).

### Variables

#### CFS/ME in the specialist services cohort

CFS/ME was defined in accordance with Centers for Disease Control and Prevention (CDC) criteria (until 2007) or National Institute for Health and Care Excellence (NICE) guidance (from 2007 onwards). These criteria are broadly similar, however, CDC criteria specify symptom duration of 6 months compared with 4 months in the NICE guidelines.

#### CFS/ME in the ALSPAC cohort

Age 13 years: Our method for defining chronic disabling fatigue at 13 years has been described previously.^14^ In brief, we identified adolescents reported by their mothers to have experienced fatigue lasting >6 months that was associated with absence from full-time school or that had prevented them from taking part in activities ‘quite a lot’ or ‘a great deal’. We excluded those whose mothers thought that the fatigue was caused by playing too much sport, who snored often and who had other illnesses that could cause fatigue (based on self-reported medication use).

Age 16 years: Our method for defining CFS at age 16 years has been described previously.^15^ Briefly, a classification of CFS/ME was based on both parental and child-reported data. We classified adolescents as chronically fatigued if parents had reported that they had fatigue lasting >6 months that had stopped them from taking part in activities ‘quite a lot’ or ‘a great deal’, that was not due to playing too much sport, and that had resulted in any absence from school/college in the past year due to tiredness or lack of energy. The child-reported data which was used in order to classify CFS/ME was based on the Chalder Fatigue Questionnaire, with a score of ≥19 (out of 33) representing CFS/ME.

#### Obesity

In the cohort of adolescents attending the specialist CFS/ME service (NOD), height and weight were measured in the clinic or GP surgery or obtained from parental report.

In the ALSPAC cohort, adolescents were invited to research clinics when they were aged approximately 13 years and 16 years. Height was measured to the last complete millimetre using the Harpenden stadiometer and weight measured using the Tanita body fat analyser (Model TBF 305). Body mass index (BMI) (kg/m^2^) was then calculated as weight/height^2^.

BMI was converted into sex-specific and age-specific Z-scores relative to the International Obesity Task Force cut-offs. These Z-scores were used to classify children into the recognised BMI categories (underweight, normal weight, overweight, obese), based on published BMI Z-score cut-offs^16^ (eg, BMI Z-score >2.288 and Z-score >2.192 are used to classify boys and girls, respectively, as obese).

### Statistical analysis

Analyses were based on differences between three groups: those with CFS/ME attending the specialist service; healthy adolescents participating in ALSPAC; and those classified as CFS/ME in ALSPAC. Across these three groups, differences in BMI and BMI Z-scores were investigated using analysis of variance tests, while the likelihood of having obesity was investigated using logistic regression. All analyses were conducted in Stata/MP V.13.1.

#### Multiple imputation to address missing data

Performing only complete-case analyses (ie, ignoring those adolescents with missing values for CFS, weight or covariates) could result in bias and would result in inflation of SEs. If missingness is dependent only on observed data (referred to as missing at random), then multiple imputation (MI) can be used to correct this bias.

##### MI in the ALSPAC cohort

We generated 99 imputed data sets based on classification of CFS (measured at 13 years and 16 years), BMI at 10 years, 11 years, 12 years, 13 years and 16 years, BMI Z-scores at 13 years and 16 years, maternal age at delivery, maternal education, total Family Adversity Index (FAI) score recorded during pregnancy (derived from responses to questions about adversity and socioeconomic status, answered by the mother, yielding scores from 0 to 18, with a higher score representing greater adversity), total FAI score recorded when the child was 8–10 years old, three additional components of the FAI recorded at 8–10 years, total unauthorised and authorised absences during year 11 of schooling (eg, one absence equates to one half-day session (two registers per day) from a typical total of 390 sessions during year 11), mean Key Stage 2 mark and total score on the Strength and Difficulties Questionnaire measured when the child was 11 years (descriptions of these and some general information on the imputation process are provided in the online supplementary material).

10.1136/archdischild-2016-311293.supp1 Supplementary material

##### MI in the specialist service data

Similarly, we generated 99 imputed data sets for the imputation of the BMI Z scores. Imputation was based on sex, score on the Hospital Anxiety and Depression Scale depression and anxiety subscales, and scores on the EQ5D.

Multivariable imputation was performed using the univariate chained equations method paired with regression switching^17^ (using Stata's user-written ‘ice’ command), combining estimates using Rubin's rules.^18^

## Results

The sample size after imputation in the specialist service cohort was 1685, representing adolescents who attended 14 specialist services between 2004 and 2015, who received a clinical diagnosis of CFS/ME and who also had complete gender data (n=1 with missing gender). The sample in the ALSPAC cohort after imputation was 13 978, which represents those who were in the ‘core’ ALSPAC sample, who were alive at 1 year and who were either a singleton or twin.

Table 1 provides descriptive statistics for the variables included in the imputation models, stratified by those classified as having CFS/ME or not at 13 years and 16 years (limited to only those in the ALSPAC cohort who had this data). In those with CFS/ME at 13 years, there was a higher proportion of boys compared with those without CFS/ME, although the CI for the difference between the two straddled 0. The opposite was apparent at 16 years. Authorised absences during year 11 of school was higher in those classified with CFS/ME compared with those classified as non-CFS/ME at 13 years (difference in means: 11.19 half-days/year; 95% CI 4.01 to 18.36) and to a greater degree in those classified as CFS/ME at 16 years (difference in means: 21.83 half-days/year; 95% CI 14.72 to 28.94). Those classified as CFS/ME at 13 years also had a higher level of life difficulties at age 11 years, as measured by the Strengths and Difficulties Questionnaire (difference in means: 6.30; 95% CI 3.61 to 8.90). There were no differences in maternal age at delivery and proportion of those with mothers educated to degree level, between the two groups.

**Table 1:** Descriptive statistics of those classified as CFS and those classified as non-CFS in ALSPAC at age 16 years

	Non-CFS ALSPAC 13 years	CFS ALSPAC 13 years	Difference (*CFS–non-CFS*) (95% CI)
Sex (male)	51.57% (50.72% to 52.42%)	56.50% (45.14% to 67.85%)	4.92% (−6.63% to 16.48%)
Total authorised absences*	20.68 (20.21 to 21.15)	31.86 (23.67 to 40.06)	11.19 (4.01 to 18.36)
Total unauthorised absences*	10.42 (9.89 to 10.96)	15.97 (5.68 to 26.26)	5.55 (−4.35 to 15.45)
Family Adversity Index (0–18)	1.85 (−2.33 to 6.03)	2.94 (−25.79 to 31.67)	1.09 (−30.96 to 33.1)
Strength and Difficulties Score (0–40)	7.39 (7.21 to 7.56)	13.69 (10.71 to 16.68)	6.30 (3.61 to 8.90)
Maternal age at delivery (years)	27.98 (27.89 to 28.06)	29.01 (27.76 to 30.26)	1.02 (−0.23 to 2.29)
Maternal education (% with degree)	12.32% (11.75% to 12.88%)	15.88% (9.39% to 22.37%)	3.57 (−2.64 to 9.77)
	**Non-CFS ALSPAC 16 years**	** CFS ALSPAC 16 years**	**Difference (*CFS–non-CFS*)** **(95% CI)**
Sex (male)	51.81% (50.91% to 52.69%)	44.59% (32.11% to 57.07%)	−7.22% (−19.99% to 5.54%)
Total authorised absences*	20.37 (19.89 to 20.85)	42.20 (35.09 to 49.31)	21.83 (14.72 to 28.94)
Total unauthorised absences*	10.36 (9.80 to 10.96)	17.19 (6.49 to 27.89)	6.83 (−3.87 to 17.53)
Family Adversity Index (0–18)	1.87 (−2.35 to 6.08)	1.95 (−18.35 to 22.25)	0.08 (−20.22 to 20.3)
Strength and Difficulties Score (0–40)	7.42 (7.24 to 7.60)	10.34 (8.52 to 12.16)	2.92 (1.10 to 4.74)
Maternal age at delivery (years)	27.99 (27.90 to 28.07)	28.38 (27.09 to 29.67)	0.39 (−0.90 to 1.68)
Maternal education (% with degree)	12.27 (11.70 to 12.84)	16.81 (10.63 to 23.00)	4.54% (−1.40% to 10.48%)

Proportions (95% CI) for categorical variables, means (95% CI) for continuous variables. Family Adversity Index collected when the child was 8–10 years.

*Half-day sessions from a typical total of 390 sessions during year 11.

ALSPAC, Avon Longitudinal Study of Parents and Children; CFS, chronic fatigue syndrome.

Table 2 reveals the mean BMI and BMI Z-scores for the three groups, as well as the prevalence of obesity in each of the three groups. At age 13 years adolescents attending the specialist service had both a larger BMI (difference: 1.15; 95% CI 0.35 to 1.95, p=0.005) and larger BMI Z-score compared with healthy adolescents in the ALSPAC cohort (difference: 0.18; 95% CI −0.12 to 0.37, p=0.067). Adolescents attending specialist services were over two times as likely to be obese compared with healthy adolescents in ALSPAC (OR: 2.31; 95% CI 1.54 to 3.48). A similar pattern was also observed at 16 years, with those attending the specialist services having a higher BMI (difference: 0.62; 95% CI −0.97 to 2.21, p=0.44), BMI Z-score (difference: 0.30; 95% CI −0.15 to 0.76, p=0.19) and an even greater likelihood of obesity compared with healthy adolescents in the ALSPAC cohort (OR: 4.07; 95% CI 2.04 to 8.11). No difference in either BMI values or the prevalence of obesity were observed between those adolescents classified as CFS/ME in ALSPAC and those attending the specialist services, at either of the two time points.

**Table 2:** BMI, BMI Z-scores and obesity prevalence across the three CFS/ME groups

	No ALSPAC (1)	Yes ALSPAC (2)	Specialist CFS/ME services (3)	Difference or OR (3 vs 1)
	CFS/ME status at 13 years			
n	13 752	226	1685	–
BMI (mean; SE)	20.52 (0.04)	20.33 (0.37)	21.67 (0.40)	1.15 (0.35 to 1.95)
Z-score (mean; SE)	0.42 (0.01)	0.34 (0.11)	0.60 (0.10)	0.18 (−0.12 to 0.37)
Obesity prev. (n; %)	655 (4.18%)	8 (3.72%)	156 (9.28%)	OR: 2.31 (1.54 to 3.48)
	CFS/ME status at 16 years			
n	13 665	313	1685	–
BMI (mean; SE)	21.61 (0.05)	21.86 (0.40)	22.23 (0.80)	0.62 (−0.97 to 2.21)
Z-score (mean; SE)	0.46 (0.01)	0.49 (0.11)	0.77 (0.23)	0.30 (−0.15 to 0.76)
Obesity prev. (n; %)	609 (4.46)	17 (5.46)	277 (16.43)	OR: 4.07 (2.04 to 8.11)

ALSPAC, Avon Longitudinal Study of Parents and Children; BMI, body mass index; CFS, chronic fatigue syndrome; ME, myalgic encephalomyelitis.

## Discussion

To the authors' knowledge, this is the first study investigating the association between obesity prevalence and CFS/ME in adolescence. We found an increased prevalence of obesity in the patient group with CFS/ME, with an OR of 2.31 and 4.07 at 13 years and 16 years, respectively, for the presence of obesity in those adolescents attending the specialist CFS/ME services versus healthy adolescents in ALSPAC. This association was largely driven by the high prevalence of obesity (9.28% and 16.43%) in those attending the specialist services, thus representing those with CFS/ME severe enough to be referred for specialist treatment.

Previous studies investigating the association between obesity and CFS have provided conflicting findings. For example, Rusu *et al*^4^ reported no association between CFS and obesity in a large sample of Canadian adolescents and adults. In another large cohort study, Viner *et al*^1^ observed no association between the prevalence of obesity and the less severe diagnosis of persistent fatigue in 1880 adolescents. Conversely, Maloney *et al*^2^ observed a twofold increase in the likelihood of having the metabolic syndrome (including abdominal obesity) in those classified as CFS (OR: 2.12; 95% CI 1.06 to 4.23) versus controls. Van't Leven *et al*^3^ also observed this increased likelihood of obesity in those classified as having a CFS-like disorder (OR: 4.1; 95% CI 2.03 to 8.45). The fact that these studies are all of a cross-sectional design warrants caution when interpreting their findings. In particular, we are unable to reliably establish whether CFS or obesity occurred first, and are thus unable to make causal conclusions about the relationship between the two. However, a longitudinal study using data from the 1970 British Cohort Study found no association between obesity at 10 years and self-reported CFS diagnosis in the period between age 10 years and 30 years,^5^ suggesting a prior classification of obesity may not be causally associated with later CFS/ME.

We do not know why there is an association between CFS/ME and obesity in those attending clinic and while the cross-sectional design limits our ability to make causal conclusions, this association could be a result of reduced physical activity levels. For example, it has been observed that those with the CFS-like disorder were also less likely to be physically active (OR: 0.1; 95% CI 0.04 to 0.22).^3^ This finding of reduced activity levels was also found in a sample of 107 patients with CFS in the north of England, who, although exhibiting no differences in sedentary behaviour, had lower levels of time spent in moderate–vigorous physical activity (>3 METS) compared with controls.^10^ This finding of no differences in sedentary behaviour is in contrast to the study by Viner and Hotopf^5^), who found that more than 4 hours per day spent sedentary was associated with an increased likelihood of having persistent fatigue in adolescence. Despite not having physical activity data for those more who attended the specialist services, we were able to compare physical activity levels of those classified as CFS/ME and healthy adolescents in the ALSPAC cohort. Compared with healthy adolescents, those classified as CFS/ME in ALSPAC spent a greater proportion of their day, on average, in sedentary activity and this was the case at both 13 years and 16 years. Furthermore, those who were classified as having CFS/ME in the ALSPAC cohort, had a much higher number of school absences compared with healthy adolescents, which may also represent further evidence of a reduction in activity level. Importantly, this association with school attendance was observed to a greater extent, in those attending the specialist services, with over 70% of these adolescents unable to attend more than 60% of school (data not shown). This provides further support for the possibility that loss of function and reduction in activity (potentially acting along a continuum of severity of CFS/ME) may be a contributory factor between CFS/ME and obesity prevalence.

### Strengths and limitations

As mentioned previously, the cross-sectional nature of the study design limits our ability to make any causal conclusions. Furthermore, as the specialist service data are primarily a clinical data set and thus limited with regard to the amount of covariates collected and with the covariate data which were collected being different to those in ALSPAC, we were unable to adjust for a range of putative confounders of the association between CFS and obesity (eg, maternal and child depression and anxiety, socioeconomic status, sleep patterns, etc). However, the use of MI to address missing data due to loss to follow-up means we have likely removed some of the bias introduced by loss to follow-up (ie, we have shown previously that loss to follow-up is predicted by variables which are also predictive of CFS and obesity, eg, socioeconomic status). Furthermore, the use of MI allows analyses to be conducted on a much larger sample size, with the consequence of more precise parameter estimates.

## Conclusion

We observed an increased prevalence of obesity in adolescents attending a specialist service compared with healthy adolescents in a population sample of the same age. Health professionals should be aware of this association to encourage appropriate screening for obesity and its possible complications when assessing patients with CFS/ME. Further longitudinal research is required in order to identify the temporal relationship between the two, while also adjusting for potential confounders of this association.
