# Correction: Obesity in adolescents with chronic fatigue syndrome: an observational study

**DOI:** 10.1136/archdischild-2016-311293corr1

**Published:** 2020-01-22

Norris T, Hawton K, Hamilton-Shield J, *et al*. Obesity in adolescents with chronic fatigue syndrome: an observational study. *Arch Dis Child* 2017;102:35–9. doi: 10.1136/archdischild-2016-311293.

The Editor of the journal has agreed to the request from the authors that the ethics statement should be changed to improve its clarity. The new statement is as follows: ‘The CFS/ME patient data used in this study were collected as part of routine clinical practice and anonymised for the National Outcomes Database. Under the Governance Arrangements for Research Ethics Committees (September 2011), ethical review is not required for research limited to the use of previously collected, non-identifiable patient information.’

