# Supplementary material for: Obesity in adolescents with chronic fatigue syndrome: an observational study
Source: Arch Dis Child. 2016 Sep 21;102(1):35–9. doi: 10.1136/archdischild-2016-311293 (PMC5256402; doi:10.1136/archdischild-2016-311293)
Supplement: Supplementary material [file archdischild-2016-311293supp_material.pdf]

Supplementary material

Rates of missing data in variables:

Variable	Number with data (% of 13 978 for ALSPAC and % of 1685 for specialist services)
**ALSPAC data**	
CFS at 13	6720 (48.08%)
CFS at 16	5756 (41.18%)
BMI at 13	5821 (41.64%)
BMI at 16	5159 (36.91%)
Sex	13 978 (100%)
Maternal age at delivery	13 978 (100%)
Year 11 unauthorised absences	10 901 (79.99%)
Year 11 authorised absences	10 901 (79.99%)
Key Stage 2 results	10 560 (75.55%)
FAI index in pregnancy	10 136 (72.51%)
FAI index at 8-10 years	7 512 (53.74%)
BMI at 10	7 087 (50.70%)
BMI at 11	6 751 (48.30%)
SDQ at 11	6650 (47.57%)
BMI at 12	6 365 (45.54%)
**Specialist services data**	
Sex	1 685 (100%)
HADS depression subscale	1 290 (76.56%)
HADS anxiety subscale	1 288 (76.44%)
EQ5D	1 005 (59.64%)
BMI at 13	260 (15.43%)
BMI at 16	250 (14.84%)

Multiple imputation

The number of imputations required to achieve convergence of parameter estimates was determined both by checking the estimate of the Monte Carlo error (MCerror) in relation to the standard error of the coefficient being estimated and also, the fraction of missing information (FMI). Continuous variables were transformed to approximate normality before entry into the imputation models and back-transformed, where appropriate, after imputation.

The auxiliary variables below were included in the imputation models due to either their strong hypothesised association with CFS/ME, the relatedness to the missingness of CFS/ME and/or the amount of missing data in these variables themselves (considered in order to construct stable imputation models which would produce reliable estimates)

Auxiliary variables used in imputation models:

Family Adversity Index (pregnancy and 8-10 years)

A family adversity index (FAI) was derived from responses to questions asked during gestation and when the child was 8-10 years old about the following family-based factors: age of mother at first pregnancy; housing (1)adequacy; (2)basic amenities; 3)defects/damp/infestations); mother's educational qualifications; financial difficulties; relationship with partner (1)status, (2)affection and aggression, (3)physical and emotional cruelty); family (1) size; (2) problems such as child in care, not with natural mother, on at risk register); social network (1)emotional; (2) practical/financial support); maternal psychopathology (1)anxiety; (2)depression or suicide attempts); substance abuse (drugs or alcohol); and crime (1)in trouble with police; (2)convictions). These ten factors yield an index with a range of 0–18.

Life difficulties

Life difficulties have been reported by parents throughout childhood and adolescence using the Strength and Difficulties Questionnaire (SDQ). This behavioural screening tool asks about 25 attributes, some positive and others negative, divided between 5 sub-scales: emotional symptoms (5 items); conduct problems (5 items); hyperactivity/inattention (5 items); peer relationship problems (5 items); prosocial behaviour (5 items). Responses to each item are scored 0-2 thus allowing each section to be scored from 0-10. A 'total difficulties' score is calculated by adding scores for the first four subscales (excluding prosocial behaviour), yielding a total score with a range of 0 – 40. For this analysis, only the SDQ reported at 140 months (11 years 8 months) was used.

School attendance and academic attainment

The total number of authorized and unauthorized school absences during the Year 11 school year (age 15-16 years) were obtained via linkage to the National Pupil Database (NPD). NPD is a pupil level longitudinal database which matches pupil and school characteristics to pupil level attainment in England (www.gov.uk/government/collections/national-pupil-database). Schools are required to take attendance registers twice a day: once at the beginning of the morning session and once during the afternoon session. In their register, schools are required to distinguish whether pupils are present, engaged in an approved educational activity, or absent. When a day pupil of compulsory school age is absent, schools have to indicate in their register whether the absence is authorized by the school or is unauthorized. An authorized absence is an absence with school permission, including instances of absences for which a satisfactory explanation has been provided (eg, for a hospital appointment). Unauthorized absences are absences without permission from a teacher or other authorized representative of the school, including all unexplained absences. Arriving late for school (without permission), after the register has closed, is recorded as unauthorized absence. Academic attainment data has also been collected from the NPD. In this analysis, the mean Key Stage 2 (KS2) score,

calculated from the results in English, Maths and Science taken in Year 6 (10 – 11 years) was used.

Maternal age at delivery and highest educational attainment

The age of the mother was calculated from the mother's date of birth (obtained at recruitment) for specific time points. Age of mother at delivery was universally collected in the cohort. Maternal highest education qualification was collected from the 'Your pregnancy' questionnaire, which was completed at 32 weeks gestation.

Results from complete case analysis

	No ALSPAC (1)	*Yes ALSPAC (2)*	*Specialist CFS/ME services (3)*	*Difference or Odds ratio (3 vs 1)*
	**CFS/ME status_ 13 year**			
n	4679	51	260	
BMI (mean; SE)	20.25 (0.05)	20.34 (0.59)	21.31 (0.30)	1.07 (0.62 – 1.49)
Z score (mean; SE)	0.35 (0.02)	0.29 (0.17)	0.68 (0.07)	0.34 (0.21 – 0.46)
Obesity prev. (n; %)	167 (3.57%)	2 (3.92%)	24 (9.23%)	OR: 2.75 (1.75 – 4.30)
	**CFS/ME status_ 16 year**			
n	3851	50	250	
BMI (mean; SE)	21.29 (0.06)	21.55 (0.64)	21.96 (0.25)	0.67 (0.22 – 1.12)
Z score (mean; SE)	0.37 (0.02)	0.40 (0.16)	0.48 (0.07)	0.10 (-0.02 – 0.23)
Obesity prev. (n; %)	152 (3.94%)	3 (6.0%)	17 (6.8%)	OR: 1.78 (1.06 – 2.98)
